# Human Mitochondrial Cytochrome *b* Variants Studied in Yeast: Not All Are Silent Polymorphisms

**DOI:** 10.1002/humu.23024

**Published:** 2016-06-27

**Authors:** Zehua Song, Anaïs Laleve, Cindy Vallières, John E. McGeehan, Rhiannon E. Lloyd, Brigitte Meunier

**Affiliations:** ^1^ Institute for Integrative Biology of the Cell (I2BC), CEA, CNRSUniversité Paris‐SudUniversité Paris‐SaclayGif‐sur‐Yvette, Cedex91198France; ^2^ Molecular Biophysics LaboratoriesInstitute of Biomedical and Biomolecular ScienceSchool of Biological SciencesUniversity of PortsmouthPortsmouthUK; ^3^ Brain Tumour Research CentreInstitute of Biomedical and Biomolecular ScienceSchool of Pharmacy and BiomedicineUniversity of PortsmouthPortsmouthUK

**Keywords:** MT‐CYB, mitochondrial DNA, yeast model, clomipramine, atovaquone

## Abstract

Variations in mitochondrial DNA (mtDNA) cytochrome *b* (*mt‐cyb*) are frequently found within the healthy population, but also occur within a spectrum of mitochondrial and common diseases. *mt‐cyb* encodes the core subunit (MT‐CYB) of complex III, a central component of the oxidative phosphorylation system that drives cellular energy production and homeostasis. Despite significant efforts, most *mt‐cyb* variations identified are not matched with corresponding biochemical data, so their functional and pathogenic consequences in humans remain elusive. While human mtDNA is recalcitrant to genetic manipulation, it is possible to introduce human‐associated point mutations into yeast mtDNA. Using this system, we reveal direct links between human *mt‐cyb* variations in key catalytic domains of MT‐CYB and significant changes to complex III activity or drug sensitivity. Strikingly, m.15257G>A (p.Asp171Asn) increased the sensitivity of yeast to the antimalarial drug atovaquone, and m.14798T>C (p.Phe18Leu) enhanced the sensitivity of yeast to the antidepressant drug clomipramine. We demonstrate that while a small number of *mt‐cyb* variations had no functional effect, others have the capacity to alter complex III properties, suggesting they could play a wider role in human health and disease than previously thought. This compendium of new *mt‐cyb*‐biochemical relationships in yeast provides a resource for future investigations in humans.

## Introduction

**Figure 1:**
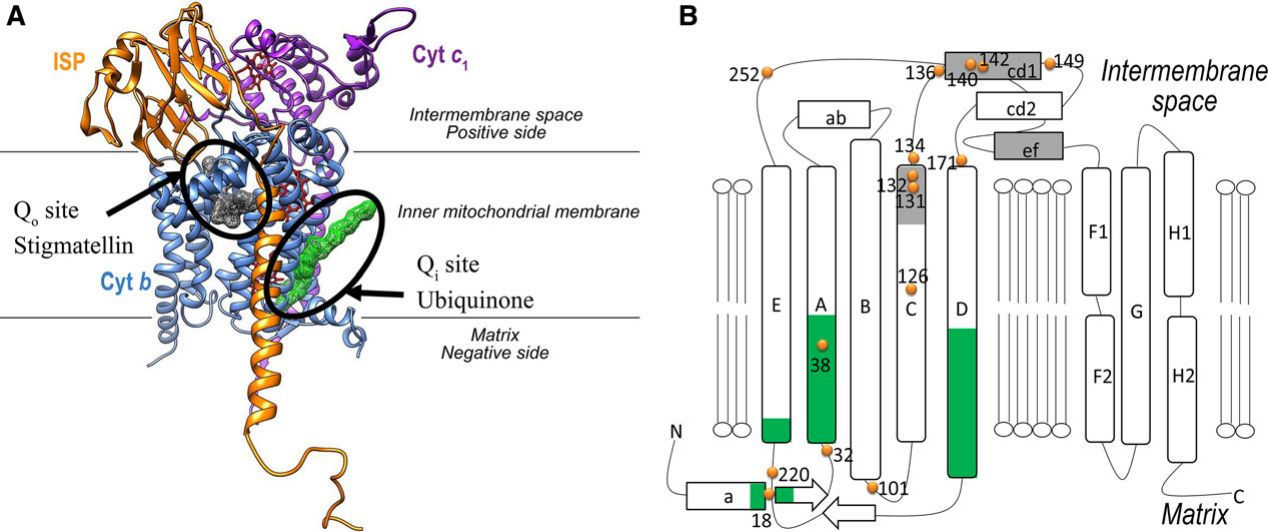
The catalytic core of yeast complex III. **A**: Redox active groups are located within three subunits that form the catalytic core: cytochrome *c_1_* (purple), the iron–sulfur protein/ISP (yellow), and cytochrome *b* (blue), which contains two b‐type hemes (*b*_l_ and *b*_h_) and forms the two quinol binding sites: Q_o_ (site of quinol oxidation), with bound stigmatellin (gray) and Q_i_ (site of quinone reduction) with bound ubiquinone (green), located on opposite sides of the membrane. During catalytic turnover, a quinol molecule binds at the Q_o_ site is deprotonated, and transfers one electron through the [2Fe‐2S] cluster of the ISP and the c‐type heme of cytochrome *c*_1_ to cytochrome *c*. Following a bifurcated pathway, a second electron is transferred across the membrane to hemes *b*_l_ and _h_ and delivered to quinone bound at the Q_i_ site, forming a stable semiquinone. A second quinol oxidation event at the Q_o_ site completes the Q‐cycle with the formation of fully reduced quinol at the Q_i_ site. In the overall reaction, two molecules of quinol are oxidized to quinone at the Q_o_ site and one molecule of quinone is reduced to quinol at the Q_i_ site with the release of four protons to the positive side of the membrane and the uptake of two protons from the matrix. The figure was drawn using the coordinates 2IBZ of yeast complex III. **B**: Schematic presentation of cytochrome *b* and location of the residues of interest. The Q_i_ region is in green; the Q_o_ region is in gray. The helices are marked by letter (A–H). The positions of the residues (human numbering) studied here are shown by orange dots.

Complex III (or b*c*_1_ complex) of the mitochondrial respiratory chain is central to the cellular energy production process. The complex is anchored within the inner mitochondrial membrane and catalyzes the transfer of electrons from ubiquinol to cytochrome *c* and couples this electron transfer to vectorial proton translocation across the inner mitochondrial membrane. The enzyme exists as a functional dimer, consisting of 10 or 11 polypeptides per monomer. All of the subunits are encoded by the nuclear genome, except cytochrome *b* (MT‐CYB; MIM# 516020), which is encoded by mitochondrial DNA (mtDNA). MT‐CYB is predominantly a hydrophobic protein consisting of eight transmembrane helices. The subunit contains two hemes and forms the two ubiquinol and inhibitor binding sites, called Q_o_ and Q_i_ sites (Fig. 1A). Complex III is a main site of proton gradient generation, thus of energy conservation. It is also a main site of reactive oxygen species (ROS) production. ROS are involved in signaling pathways that coordinate both the nucleus and mitochondria to drive beneficial homeostatic responses, but they can also damage cellular components that induce cell death.

In the human population, the *mt‐*cyb exhibits a high level of variation. Over 410 missense changes from the *Homo sapiens* mitochondrion complete genome reference sequence (rCRS: GenBank NC_012920.1) have been reported (http://mitomap.org) and 90 in MSeqDR‐LSDB (https://mseqdr.org/MITO/genes/MT-CYB), with some overlap between the various databases.

While a number of these variations are likely to be silent, some variations, especially those located in regions involved in the catalytic activity and inhibitor binding, may induce subtle changes in complex III function. Under exposure to inhibitors, to different energy substrates or to higher energy demands, the altered function of the variant complex III might be revealed, which could affect the fitness of cells and the health of the individuals bearing these variants. Thus, what may have previously been considered a silent variation, can, under different physiological circumstance, result in a significant effect. Indeed, a recent analysis of 874 genes in 589,306 genomes identified 13 adults harboring highly penetrant mutations that typically cause eight severe Mendelian conditions, with no reported clinical manifestations [Chen et al., 2016], which further suggests that “silent,”‐“nonsilent” mutation paradigm is questionable. Several studies have addressed the question of the consequences of *mt‐cyb* variations at a population level. For example, m.15257G>A that causes a D to N substitution at position 171 (p.Asp171Asn) of the MT‐CYB polypeptide [Heher and Johns, 1993; Johns and Neufeld, 1993; Johns et al., 1993] is associated with Leber hereditary optic neuropathy (LHON) and a number of different disease cohorts, whereas m.14798T>C that causes a phenylalanine to leucine amino acid substitution in MT‐CYB (p.Phe18Leu) is frequently found in patient‐derived glioblastoma biopsy cells [Kirches et al., 2001; Larman et al., 2012; Lloyd et al., 2015], but biochemical data are often lacking. Obtaining such biochemical data is experimentally challenging due to the absence of tools for introducing single variations into mammalian mtDNA. This hiatus in these data could either lead to potentially important mtDNA variations in health and disease being incorrectly dismissed, or to finite resources being erroneously diverted toward the investigation of those that are not important.

Yeast (*Saccharomyces cerevisiae*) is a convenient model for the study of point mutations in the core subunit of complex III. The organism has well‐known advantages. Cells can survive in the absence of respiration relying exclusively on fermentation as an energy source. Therefore, the severe malfunction or even the absence of the respiratory enzymes is not lethal. Yeast complex III, and especially the catalytic core, is very similar to the mammalian enzyme, and several atomic structures are available. Many genetic tools are well developed. Of particular interest is the mitochondrial transformation technique by which chosen mutations can be introduced into mitochondrially encoded genes, such as *mt‐cyb*. So far, only *S. cerevisiae* and the green alga *Chlamydomonas reinhardtii* are amenable to mitochondrial transformation. Mitochondrial mutants can then be analyzed by a broad range of biochemical/biophysical methods. Using that approach, we have previously assessed the impact of human disease mutations on complex III and used mutants with severe respiratory function defect to explore possible compensation mechanisms [Fisher et al., 2004a, 2004b; Meunier et al., 2012]. The yeast model is also an easy‐to‐use tool for characterizing acquired resistance mutations reported in *mt‐cyb* of pathogens after treatment by drugs targeting complex III or to explore the structural basis of the natural differential sensitivity of complex III to inhibitors [Hill et al., 2003; Song et al., 2015a, 2015b].

Here, taking advantage of the yeast model, we studied 22 single human variations, including some with reported disease associations, to obtain biochemical information on their functional impact. We focused on monitoring the effect of mutations that cause nonsynonymous amino‐acid substitutions located in, or in the vicinity of, the MT‐CYB catalytic/binding domains (Fig. 1B). In particular, we tested the impact of frequent polymorphisms: (1) p.Phe18Leu located in the Q_i_ site on complex III sensitivity to the antidepression compound clomipramine and (2) p.Asp171Asn, the signature of haplogroup J, located nearby the Q_0_ site on the complex III sensitivity to the antimalaria drug atovaquone.

We show that some variants previously reported as “silent” mutations, including some frequent polymorphisms, significantly modify the properties of the yeast complex III, suggesting they may play a greater role in human health and disease than previously thought.

## Materials and Methods

### Data Collection

For each human mutation under investigation, information on the prevalence and heteroplasmy level in normal/healthy and pathologic tissues was obtained from the following five databases: MITOMAP (http://www.mitomap.org/MITOMAP), HmtDB (http://www.hmtdb.uniba.it/hmdb/), mtDB (http://www.mtdb.igp.uu.se/), and MSeqDR‐LSDB (https://mseqdr.org/MITO/genes/MT-CYB)) and an in‐house glioblastoma (GBM) database [Lloyd et al., 2015]. Known genotype–phenotype correlations were explored using OMIM (http://www.omim.org/).

### Media and Chemicals

The following media were used for the growth of yeast: YPD (1% yeast extract, 2% peptone, 3% glucose), YPG (1% yeast extract, 2% peptone, 3% glycerol), YPEth (1% yeast extract, 2% peptone, 3% ethanol), YPGal (1% yeast extract, 2% peptone, 0.1% glucose, 3% galactose). Equine cytochrome *c*, decylubiquinone, atovaquone, and clomipramine were purchased from Sigma–Aldrich Chimie, Lyon, Rhone Alpes, France.

### Generation of Yeast Cytochrome *b* Mutants

The plasmid pBM5 carrying the wild‐type intron‐less sequence of *mt‐cyb* gene was constructed by blunt end cloning of *mt‐cyb* PCR product into the pCRscript vector (Agilent Technologies, Les Ulis, Cedex, France). The mutagenesis was performed using the Quickchange Site‐Directed Mutagenesis Kit (Agilent Technologies) according to the manufacturer's recommendations. After verification of the sequence, the plasmids carrying the mutated genes were used for biolistic transformation. The mitochondrial transformation was performed by microprojectile bombardment as described in Hill et al. (2003) and Meunier (2001). The strains are identical except for the mutations introduced in *mt‐cyb*, and are all homoplasmic (i.e., they contain only one mtDNA population).

Diploids were used in the experiments, except for monitoring the sensitivity of respiratory growth to atovaquone, where the AD1‐9 series was used. AD1‐9 strain harbors multiple deletions in the ABC transporter genes that render the strain more sensitive to drugs than standard yeast strains (α ura3 his1, yor1Δ::hisG, snq2Δ::hisG, pdr5Δ:: hisG, pdr10Δ::hisG, pdr11Δ::hisG, ycf1Δ::hisG, pdr3Δ::hisG, pdr15Δ::hisG, pdr1Δ::hisG). We previously found that while the yeast complex III is highly sensitive to atovaquone, the drug cannot reach the complex unless ABC transporters are deactivated [Hill et al., 2003].

### Preparation of Mitochondria and Measurement of Cytochrome c Reductase Activity

Yeast mitochondria were prepared as in Lemaire and Dujardin (2008). Briefly, yeast grown in YPGal medium were harvested at mid‐log phase. Protoplasts were obtained by enzymatic digestion of the cell wall using zymolyase in an osmotic protection buffer. Mitochondria were then prepared by differential centrifugation following osmotic shock of the protoplasts. Mitochondrial samples were aliquoted and stored at −80°C. Concentration of complex III in the mitochondrial samples was determined from dithionite‐reduced optical spectra, using *ε* = 28.5 mM^−1^/cm at 562 − 575 nm. Decylubiquinol‐cytochrome *c* reductase activities were determined at room temperature by measuring the reduction of cytochrome *c* (final concentration of 20 μM) at 550 nm versus 540 nm over 1‐min time course in 10 mM potassium phosphate pH 7, 0.01% (w/v) lauryl‐maltoside and 1 mM KCN. Mitochondria were added to obtain a final concentration of 5–30 nM of complex III. Activity was initiated by the addition of decylubiquinol, a synthetic analogue of ubiquinol (final concentration of 40 μM). Initial rates were measured as a function of the decylubiquinol concentration. Each measurement was repeated three times and the values obtained were averaged. Activity (turnover) was estimated as cytochrome *c* reduction rates per complex III.

### Inhibitor Titration

Cytochrome *c* reduction activity was measured as described above in the presence of increasing concentrations of inhibitors (six to 10 different concentrations). Each measurement was repeated two or three times and averaged. The midpoint inhibition concentrations (IC_50_) were determined from the titrations and the IC_50_ values were normalized by the concentration of complex III.

### Respiratory Growth Assays

Yeast strains were grown in 5 ml of YPEth medium with increasing concentration of drug. Cultures were inoculated from 1‐day‐old cultures on YPG to an OD_600_ nm of 0.2 and incubated at 28°C with vigorous shaking to maintain good aeration. Cell densities measured as OD_600_ nm were estimated 2 or 3 days after inoculation.

## Results and Discussion

### Spectrum of Human mt‐cyb Variations Analyzed in Yeast

The 22 human polymorphisms chosen for analysis in yeast are presented in Table 1. With the exception of one variation (m.15048G>C), reported in a single GBM‐patient [Lloyd et al., 2015], all variations were reported as polymorphisms in one or more of the mitochondrial databases. Eleven polymorphisms have no known disease association: m.15122A>G, 15147C>T, 15171G>A, 15191T>A, 15258GA>AG, 15048G>C, 15404G>C, 15138A>G, 15152G>A, 15153G>A, and 15502C>G, and were rare, being found in typically 10 cases or less in the normal/healthy human population. Nine polymorphisms had one or more reported disease associations: m.15140G>A, 15164T>C, 15257G>A, 15258A>G, 14798T>C, 14841A>G, 14858G>A, 15047G>A, and 15500G>A. The top three most frequently recorded polymorphisms in normal healthy cases, were also the most frequently recorded in disease‐associated cases (in descending order of patient frequency): m.14798T>C, 15257G>A, and 15047G>A. The individual *mt‐cyb* variations were associated with one or more of the following type of disorders: cardiological (e.g., cardiomyopathy and Noonan syndrome); neurological (e.g., LHON, Parkinson's and schizophrenia); metabolic (e.g., diabetes); and cancers (e.g., breast, thyroid, GBM, pituitary adenoma, oral squamous cell carcinoma, and lung), there were no genotype–phenotype correlations in the OMIM database, with the exception of m.15257G>A, which is associated 9% of LHON cases [Heher and Johns, 1993; Johns and Neufeld, 1993; Johns et al., 1993].

**Table 1:** Prevalence of Human *mt‐cyb* Variations in the Normal and Pathologic Population and Their Predicted Effect on the Complex III Properties

Human						Yeast
Nucleotide substitution^a^	Amino‐acid substitution	Normal, healthy (number of cases)	Pathologic (disease) (number of cases)	Heteroplasmy found in human tissue (%)	Predicted effect on complex III (based on yeast model and on structure)	Matching amino‐acid substitution^b^
**Q_o_ domain: atovaquone sensitivity**						
m.15122A>G	p.Thr126Ala	5	0	n/a	None	p.Thr127Ile
m.15140G>A	p.Val132Ile	1	3 (breast and thyroid cancer)		Slight modification of ato sensitivity	CysCysVal_133–135_ ValLeuPro
m.15147C>T	p.Pro134Leu	*n*	0	n/a	Slight modification of ato sensitivity	
m.15164T>C	p.Phe140Leu	6	1 (diabetes)		Slight modification of ato sensitivity	p.His141Tyr/Phe
m.15171G>A	p.Gly142Glu	1	0	n/a	Severely decreased activity, decreased ato sensitivity	p.Gly143Ala
m.15191T>A	p.Leu149Met	2	0	n/a	Decreased ato sensitivity and activity	p.Leu150Phe
	p.Asp171 (rCRS)					p.Ser172Asp
m.15257G>A	p.Asp171Asn	319 (haplogroup J)	51^c^	100 (GBM)	Increased sensitivity to ato	p.Ser172Asn
m.15258A>G	p.Asp171Gly	24	2 (diabetes type2/LHON)		Increased sensitivity to ato	p.Ser172Gly
m.15258GA>AG	p.Asp171Ser	*n*	0	n/a	Increased sensitivity to ato	p.Ser172 (wt)
**Q_i_ domain: clomipramine sensitivity**						
	p.Phe18 (rCRS)					p.Ile17Phe
m.14798T>C	p.Phe18Leu	4,410	233^d^	99 (GBM)	Increased sensitivity to clom	p.Ile17 (wt)
m.14841A>G	p.Asn32Ser	1	1 (LHON)		Increased sensitivity to clom, decreased activity	p.Asn31Ser
m.14858G>A	p.Gly38Ser	10	2 (mental disorder, diabetes)		Increased sensitivity to clom	p.Gly37Ser
m.15048G>C	p.Gly101Ala	*n*	0	n/a	None	
m.15047G>A	p.Gly101Ser	50	3 (thyroid cancer, diabetes type II)		None	
m.15048G>A	p.Gly101Asp	0	1 (GBM)	17 (GBM)	Decreased sensitivity to clom	p.Gly100Asp
m.15404T>C and 15406C>A	p.Phe220Leu	2	0	n/a	Increased sensitivity to clom	p.Phe225Leu
**Q_o_ domain: proton pathway**						
m.15138A>G	p.Tyr131Cys	*n*	0	n/a	None or slightly decreased activity	p.Tyr132Phe
m.15152G>A	p.Gly136Ser	3	0	n/a	Severely decreased activity	p.Gly137Arg
m.15153G>A	p.Gly136Asp	4	0	n/a	Severely decreased activity	p.Gly137Glu
	p.Asp252(rCRS)					p.His253Asp
m.15502C>G	p.Asp252Glu	1	0	n/a	None	p.His253Glu
m.15500G>A	p.Asp252Asn	1	2 (diabetes/GBM)	5 (GBM)	None	p.His253Asn

n, undetermined number of cases.

n/a, not applicable.

^a^Nucleotide variations are relative to the *Homo sapiens* mitochondrion complete genome (GenBank NC_012920.1).

^b^Mutations were introduced in yeast cytochrome *b* using the mitochondrial transformation technique (*Materials and Methods*) with the exception of G137E that had been obtained by random mutagenesis.

^c^GBM, pituitary adenoma, Parkinson's, CADASIL, Noonan syndrome, schizophrenia, LHON, oral squamous cell carcinoma, lung cancer.

^d^GBM, thyroid tumors, LHON, prostate cancer, type I endometrial carcinoma, schizophrenia, breast cancer, cardiomyopathy, optic atrophy, CPEO, pituitary adenoma, oral cavity carcinoma, Warthin tumor, OXPHOS system deficiency, Noonan syndrome.

The polymorphisms were chosen on the basis of the location of the residues in the structure of complex III. Nine variations (at positions 126, 132, 134, 140, 142, 149, and 171) are located in or nearby the Q_o_ site. Eight variations (at positions 18, 32, 38, 101, and 220) are in or nearby the Q_i_ site. Five variations (at positions 131, 136, and 252) are located in possible proton routes from the Q_o_ site (Table 1; Fig. 1B).

### Mutations Within or Nearby the Q_o_ Site Influence Atovaquone Sensitivity

Atovaquone (used in combination with proguanil, and marketed as Malarone®) is a popular prophylactic drug that also shows high efficiency in the treatment of uncomplicated *Plasmodium falciparum* malaria. The compound is also used to treat *Pneumocystis jirovecii* pneumonia, *Toxoplasma gondii* toxoplasmosis, and other infections. Atovaquone inhibits complex III by binding in the Q_o_ site, as revealed by the resolution of the atomic structure of yeast complex III with bound atovaquone (PDB code 4PD4) [Birth et al., 2014] and by the Q_o_ site location of point mutations found in the *mt‐cyb* of atovaquone‐resistant parasites [Hill et al., 2003; Mather et al., 2005].

The complex III of the malaria parasite—and yeast—is highly sensitive to atovaquone, whereas the mammalian complex III is naturally more resistant to atovaquone, which is expected of an antimalarial compound used to treat humans. The IC_50_ (midpoint inhibition concentration) values per nM complex III were estimated to be 75 and 4 nM for the bovine and the yeast enzymes, respectively [Vallières et al., 2013]. The IC_50_ value for the malaria parasite enzyme was reported to be similar to the yeast value, around 3 nM [Biagini et al., 2008]. The differential sensitivity between mammalian and *Plasmodium* (and yeast) is likely to be due to amino‐acid variations in the Q_o_ site, more specifically, variations of MT‐CYB residues 275, 278, and 295, as suggested by mutational analysis using the yeast model [Vallières et al., 2013].

**Figure 2:**
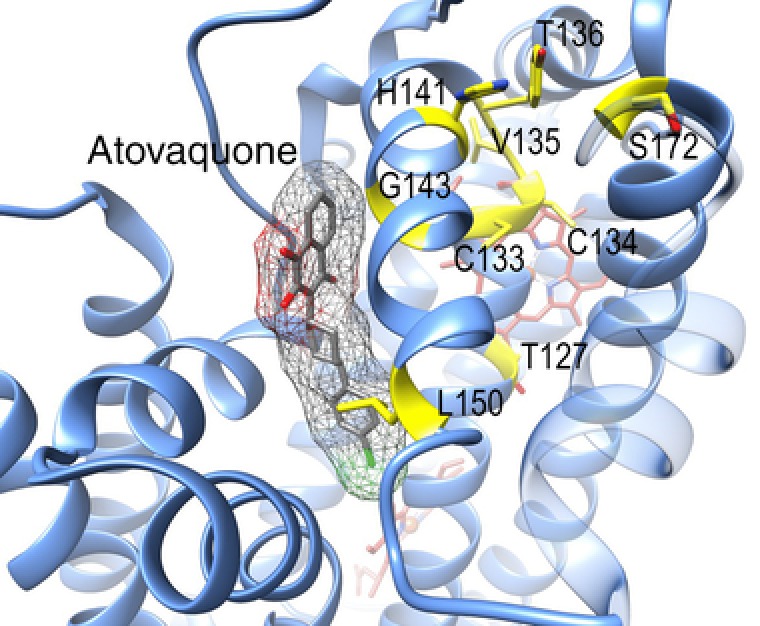
Location of the residues in yeast Q_o_ domain with bound atovaquone. Atovaquone (gray) is bound is a position similar to stigmatellin (Fig. 1A). Heme *b*_l_ is in red. Yeast numbering is used. Residues p.Gly143, p.Leu150, p.Thr127, p.Cys133, p.Cys134, p.Val135, p.Thr136, p.His141, and p.Ser172 are colored in yellow and their sidechains are shown. The figure was drawn using the coordinates 4PD4 of yeast complex III.

Nine of the chosen human polymorphisms (Table 1) are located at seven positions in or near the atovaquone binding pocket, and thus might modify the sensitivity of the enzyme to the drug (Fig. 2). In order to explore this, we generated yeast mutants harboring equivalent MT‐CYB mutations and analyzed their atovaquone sensitivity; we also reviewed previously published data.

The human p.Gly142Glu (yeast p.Gly143) and p.Leu149Met (yeast p.Leu150) (Table 1) are located in the atovaquone binding site (Fig. 2). We predict that these substitutions would alter the sensitivity to atovaquone but also interfere with catalytic activity, by hindering the correct binding of the substrate ubiquinol. In yeast, mutation p.Gly143Ala—a minor addition of a single carbon atom compared with the addition of a bulky acidic group in p.Gly143Glu—confers a ninefold increase in atovaquone resistance [Fisher and Meunier, 2005] (Supp. Table S1). p.Gly143Asp was studied in a bacterial complex III and was shown to decrease enzymatic activity severely [Daldal et al., 1989] (Supp. Table S1). It is likely that in human complex III, p.Gly142Glu would also result in an inactive enzyme. Mutation p.Leu149Met would have a less dramatic effect on the enzyme activity. In yeast, mutation p.Leu150Phe results in a threefold increase in atovaquone resistance with the loss of 50% of complex III activity [Hill et al., 2003] (Supp. Table S1).

Human variants p.Val132Ile, p.Pro134Leu, and p.Phe140Leu (Table 1) are located in the vicinity of the Q_o_ pocket (residues 133, 135, 141 in yeast [Fig. 2]). We have previously observed that, in yeast, atovaquone sensitivity was modified by mutations in the region 133–141 (Supp. Table S1). The residues are not in direct contact with bound atovaquone in the crystal structure but mutations could indirectly modify the binding pocket and, by consequence, slightly alter the binding of the drug [Fisher and Meunier 2005; Vallières et al., 2013]. p.Thr126Ala is located distal from the Q_o_ binding pocket. As expected, the matching yeast mutation p.Thr127Ile had no effect on complex III activity and atovaquone sensitivity [Hill et al., 2003] (Supp. Table S1).

We then sought to determine whether mutations of human residue 171 (172 in yeast) (Table 1), also located in the vicinity of the Q_o_ site (Fig. 2), could modify the sensitivity to atovaquone.

**Figure 3:**
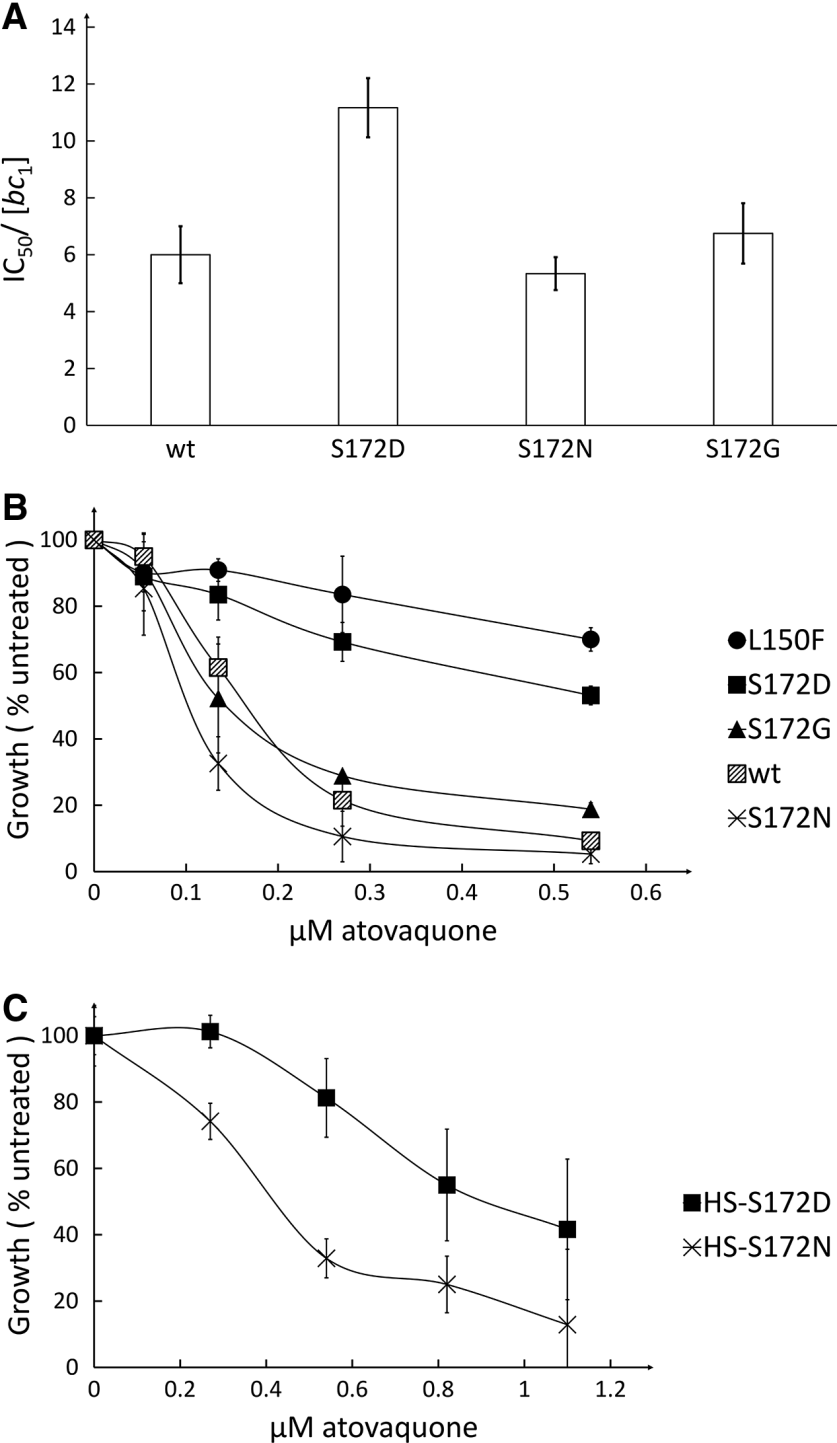
**A**: Atovaquone sensitivity of yeast mutant and wild‐type control complex III. IC_50_s, midpoint inhibition concentrations, were estimated from the titration measurements and reported per complex III (*Materials and Methods*). Each measurement was repeated three times and averaged. Error bars represent standard deviations. **B** and **C**: Sensitivity of yeast mutant and wild‐type control respiratory growth to atovaquone. Atovaquone sensitivity is presented for each strain as the percentage of growth relative to wild‐type control, that is, untreated. Each measurement was repeated at least twice and averaged. Error bars represent standard deviations. **B**: Strain harboring single mutation p.Ser172Asp, Asn, Gly and p.Leu150Phe. **C**: Strain with multiple changes (HS) in the Q_o_ site that replace yeast residues by human equivalents.

The yeast residue, Ser172 (wt), which also reflects a rare polymorphism in humans, was replaced by Asp, the residue present in the human reference sequence; Asn, the frequent polymorphism and marker of haplogroup J in humans; and finally Gly, also a polymorphism in humans. Modified IC_50_ values to atovaquone were observed (Fig. 3A). Complex III with p.Ser172Asp was found to be more resistant (IC_50_ of 11.5) than the wt, Gly, and Asn (IC_50s_ of 7, 7.5, and 5, respectively) to atovaquone. None of the mutations had a major effect on the catalytic activity of the complex (Supp. Table S1). We then tested whether these small changes in sensitivity could be observed when monitoring the respiratory growth of mutants and control strains. As shown in Figure 3B, p.Ser172Asn, p.Ser172Gly, and the wt displayed an increased sensitivity compared with p.Ser172Asp (and to p.Leu150Phe, mentioned above).

In order to confirm the differential sensitivity induced by the polymorphisms at position 172, we tested whether the same behavior could be observed in mutant strains that harbor a complex III Q_o_ site that more closely resembled that of the human enzyme. To this end, two strains were genetically constructed. In addition to the amino acid substitution at position 172, eight mutations in the Q_o_ site were introduced by replacing the yeast residues by their human equivalents: CysCysVal_133–135_ ValLeuPro, p.His141Phe, p.Ley275Phe, p.Phe278Ala, and p.Met295Leu. The resulting strains, called HS‐p.Ser172Asp and HS‐p.Ser172Asn, were tested for the sensitivity to atovaquone (Fig. 3C). Both strains were inhibited at slightly higher doses of atovaquone than the control strain. This was expected as we have previously observed that yeast to human amino acid substitutions at positions 275, 278, and 295 resulted in an increased resistance to the drug [Vallières et al., 2013]. Despite this, HS‐p.Ser172Asn was still more sensitive to atovaquone than HS‐p.Ser172Asp.

### Mutations Within and Nearby the Q_i_ Site Modify Clomipramine Sensitivity

The main clinical use of clomipramine is as a tricyclic antidepressant, but it is also showing promise in preclinical studies for the treatment of glioma, potentially via a mitochondrial targeted mechanism. In addition to its main mode of action, which is to bind nonselectively to 5‐hydroxytryptaimine and noradrenaline uptake receptors, which endows it with antidepressant activity [Rang et al., 2011], the drug has been shown to induce apoptosis in malignant glioma via the direct release of cytochrome *c* that stimulates the p‐c‐Jun pathway and triggers the caspase cascade [Levkovitz et al., 2005; Pilkington et al., 2008]. Furthermore, clomipramine has been shown to impair cell respiration and inhibit complex III in glioma, thus implicating the complex III in the glioma cell killing effect of this drug [Daley et al., 2005; Higgins and Pilkington, 2010].

**Figure 4:**
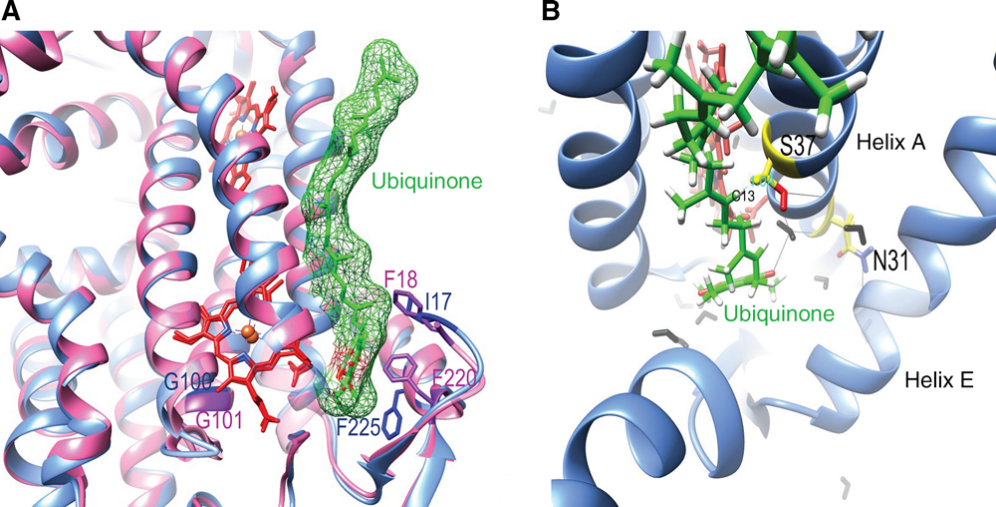
**A**: Comparison of the yeast and mammalian Q_i_ sites. Superposition of yeast (blue) and bovine (pink) cytochrome *b* structures. Ubiquinol is in green and heme *b*_h_, in red. The figure was drawn using the coordinates 4PD4 and 1PP9 of yeast and bovine complex III, respectively. The two structures were superposed using Chimera. Residues p.Ile17, p.Gly100, and p.Phe225 of yeast cytochrome *b* and p.Phe220, p.Gly101, and p.Phe220 of mammalian cytochrome *b* are shown. **B**: Possible interactions between p.Asn31 and helix E, and between p.Gly37Ser and ubiquinol. Cytochrome *b* is colored in blue, ubiquinol in green, p.Asn31 and p.Ser37 in yellow, waters as black sticks, and heme *b*_h_ in red. Possible hydrogen bonds are indicated in black lines. Minimum distance between p.Ser37 and ubiquinol p.Cys13 hydrogen is 1.0 Å. The structure was drawn using 2IBZ. In silico mutation p.Gly37Ser was introduced using Chimera.

We tested clomipramine inhibitory effect on the catalytic activity of bovine and yeast complex III, using purified mitochondria, and determined the IC_50_s. The drug was found to be a weak inhibitor of the enzymes, with IC_50_ values of 24 ± 5 and 3.4 ± 0.1 μM per nM of bovine and yeast complex, respectively. Bovine complex III seems, thus, naturally less sensitive to clomipramine than the yeast enzyme. Complex III possesses two distinct inhibitor binding sites, the Q_o_ (that binds atovaquone) and the Q_i_ site. Comparisons of the bovine and yeast Q_i_ sites reveal structural differences that could be responsible for the observed differential sensitivity to clomipramine, in particular the replacement of p.Ile17 present in yeast by Phe present in the mammalian enzyme (Fig. 4A). Intriguingly, we have previously identified a parallel amino acid substitution (p.Phe18Leu) in 30% of biopsy‐derived GBM samples, and predicted it to affect complex III inhibitor binding [Lloyd et al., 2015].

We thus sought to determine whether variations in the Q_i_ site could influence sensitivity of complex III to clomipramine.

**Figure 5:**
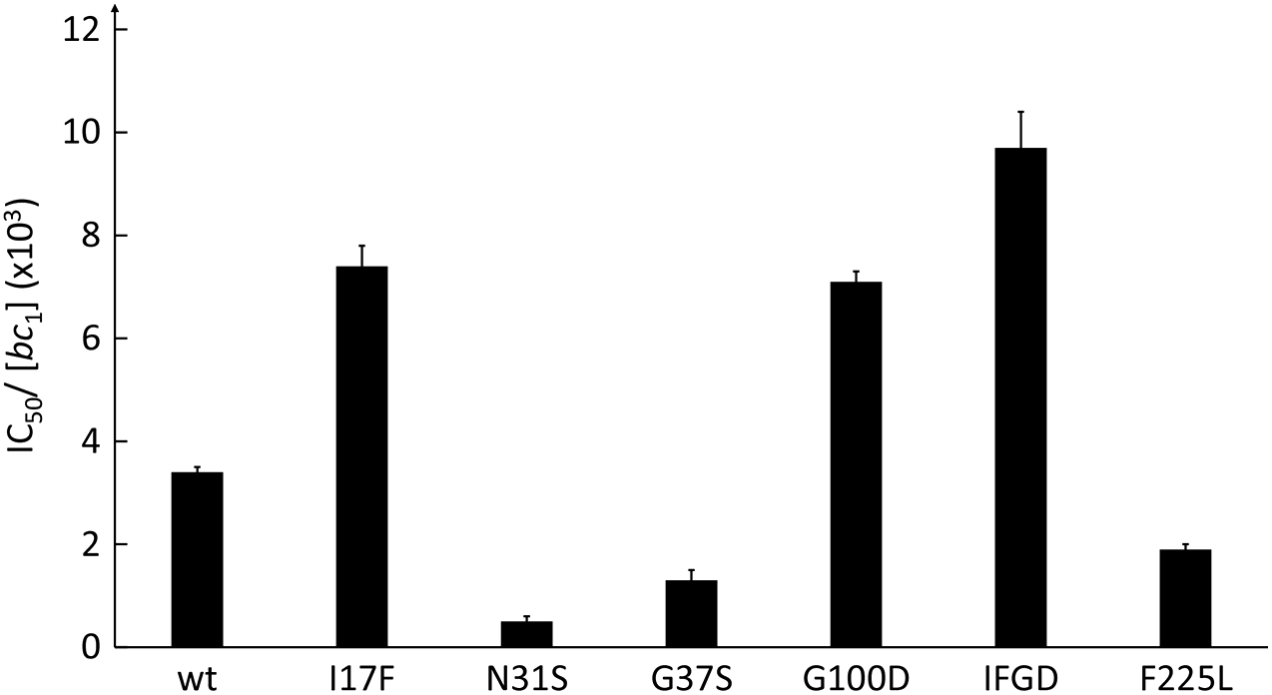
Clomipramine sensitivity of yeast mutant and wild‐type control complex III. IC_50_s, midpoint inhibition concentrations, were estimated from the titration measurements and normalized for complex III concentration (*Materials and Methods*). Each measurements was repeated three times and averaged. Error bars represent standard deviations. IFGD: p.Ile17Phe+p.Gly100Asp.

As shown in Figure 5, the mutation p.Ile17Phe (replacing the yeast amino acid by the human residue) resulted in a twofold decrease in clomipramine sensitivity. Thus, the phenylalanine naturally present at that position (p.Phe18) in mammalian MT‐CYB could explain, at least in part, the decreased clomipramine sensitivity of the mammalian enzyme compared with the yeast enzyme. The frequent polymorphism p.Phe18Leu in humans corresponding to yeast wild‐type p.Ile17 is likely to result in an increase sensitivity to the drug.

Other mutations in or in the vicinity of the Q_i_ site were also investigated: p.Asn31Ser (p.Asn32Ser and rare polymorphism in humans), p.Gly37Ser (p.Gly38Ser and a rare polymorphism in humans) (Fig. 4B), p.Gly100Asp (p.Gly101Asp reported in a GBM‐patient) [Lloyd et al., 2015], and p.Phe225Leu (structurally similar to p.Phe220Leu in humans, rare polymorphism). p.Gly100Asp resulted in decreased clomipramine sensitivity, whereas p.Asn31Ser, p.Gly37Ser, and p.Phe225Leu increased the sensitivity to the drug (Fig. 5). Interestingly, the double mutation p.Ile17Phe‐p.Gly100Asp had an additive effect, further decreasing clomipramine sensitivity. These data substantiate the hypothesis that Q_i_ site is a clomipramine target and Q_i_ site mutations can affect clomipramine sensitivity. None of these mutations had a major impact on complex III activity, except p.Asn31Ser that resulted in a twofold decrease: its activity was 25 ± 0.8 sec^−1^ compared with 56 ± 4.6 sec^−1^ for the wild type (Supp. Table S1).

The examination of the structure of the Q_i_ pocket domain (Fig. 4A) showed that replacement of the small residue p.Ile17 by a bulky phenylalanine (p.Ile17Phe) could sterically hinder the binding of clomipramine, which is a far larger molecule than the natural ligand, whereas the substitution of F225 by the smaller leucine (p.Phe225Leu) would leave more space for the binding of the drug.

The effect of p.Gly100Asp is less straightforward to explain as the residue is not located directly in Q_i_ site. However, the replacement of the small glycine by the bigger and charged aspartate might indirectly alter the pocket reducing its affinity for the drug. That major change (p.Gly100Asp) had minimal effect on the catalytic activity of the complex III. As the enzyme seems to accommodate p.Gly100Asp, it is likely that the less drastic substitutions reported in humans, p.Gly101Ala and p.Gly101Ser, would also have minimal impact on the enzyme properties.

The increased clomipramine sensitivity induced by p.Asn31Ser and p.Gly37Ser might be caused by modifications of the binding pocket (Fig. 4B). As we have previously reported, the equivalent residue to p.Asp31 in human (p.Asp32) participates in stabilizing hydrogen bonds with key residues in the Q_i_ site [Lloyd and McGeehan, 2013]. The introduction of a serine might slightly modify the local structure, which would stabilize clomipramine but decrease the catalytic activity. A serine at position 37 might slightly tighten the drug binding without impairing the activity.

### Q_o_ Proton Pathway Mutations Influence Complex III Activity

As explained in Figure 1, during the catalytic reaction, a quinol is oxidized in the Q_o_ site. Upon oxidation, two protons are released in the solvent phase at the intermembrane side of the inner membrane. Several residues and water molecules are likely to form pathways for proton movement [Hunte et al., 2003; Palsdottir et al., 2003]. The conserved residue p.Glu272 seems to be one of the main actors in the proton relay [Wenz et al., 2006; Seddiki et al., 2008; Crofts et al., 2013]. P.His253 and p.Tyr132 also interact with water molecules and might be involved in forming proton routes. P.Gly137, in contact with the conserved p.Ser140, is located in the vicinity of this water‐polar residue network (Supp. Fig. S1). We hypothesize that mutation of these residues could impair the proton movement and by consequence the catalytic activity of complex III.

**Figure 6:**
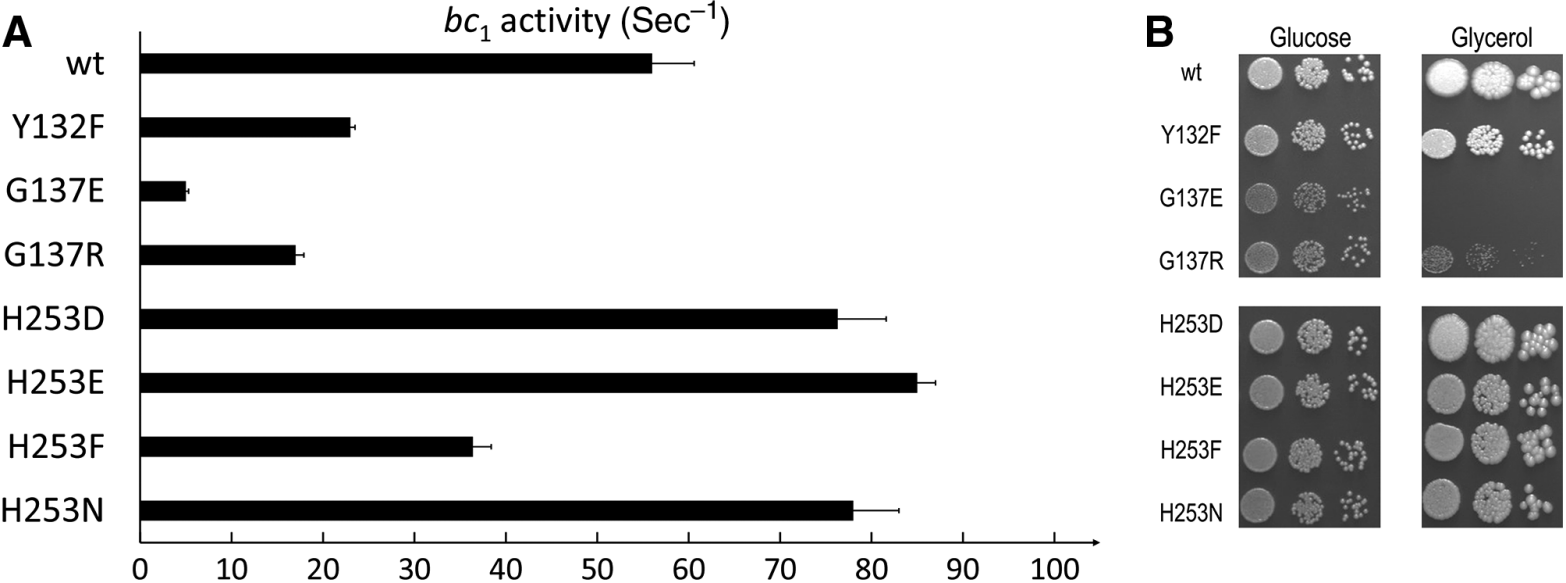
**A**: Complex III activity in wild‐type control and mutants. The ubiquinol cytochrome *c* reductase assays were performed as described in *Materials and Methods*. The activities measured at saturated substrate concentrations (40 mM decylubiquinol) were reported relative to complex III concentration. The measurements were repeated at least twice and averaged. The error bars represent standard deviations. **B**: Respiratory growth of wild‐type control and mutants. Serial dilutions in water of cells pregrown on glucose plates were spotted on plates containing either glucose (YPD, fermentative medium) or glycerol (YPG, respiratory medium) and incubated for two (YPD) to 4 days (YPG) at 28°C.

In order to test this hypothesis systematically, we replaced p.His253 in yeast by Asp (human rCRS), Glu (reported as a polymorphism), and Asn (reported polymorphism and in GBM) (Table 1). His, Asp, Glu, and Asn are polar residues. In parallel, we tested the effect of introducing the apolar residue Phe at that position, even though that mutation has not been observed in humans. We also assessed the effect of replacing the polar residue Y132 by the apolar Phe. Two other mutations were tested, p.Gly137Glu and p.Gly137Arg, corresponding to the human polymorphisms, p.Gly136Asp and p.Gly136Ser. We measured the complex III activity and monitored the respiratory growth of the yeast mutants (Fig. 6).

Replacement of p.His253 by Phe decreased the electron transfer activity of the complex by 40% (Fig. 6A). The introduction of the human residues Asp, Glu, or Asn resulted in a complex with a higher electron transfer activity (130%–160% of the control activity). The introduction of an apolar residue at position 253 probably slows down proton movement and concomitant electron transfer. Asp, Glu, or Asn might facilitate the proton translocation and thus increase the electron transfer rate. In organisms with a nonprotonable residue at position 272, a glutamate at position equivalent to p.His253 was observed, suggesting a role in proton movement [Brasseur et al., 2002].

The mutations of p.His253 had no effect on the respiratory growth competence (Fig. 6B). The substitution p.Tyr132Phe had a more severe impact and decreased complex III activity by around 50% (Fig. 6A) resulting in a lower respiratory growth competence (Fig. 6B). The apolar residue Phe is likely to disrupt the water network and proton routes. Examination of the structure suggests that a polar residue is required at that position. Thus, the replacement of the tyrosine by a cysteine (human polymorphism p.Tyr131Cys) might have only a mild effect.

In contrast to p.Tyr131Cys, the human polymorphisms p.Gly136Ser and particularly p.Gly136Asp (Table 1) are expected to have dramatic effects. In yeast, the equivalent mutations p.Gly137Arg and p.Gly137Glu caused, respectively, 70% and 90% decrease in complex III activity and a very severe respiratory growth defect (Fig. 6). The introduction of charged or polar residues at that position could severely impair the transfer of protons from the Q_o_ site toward the negative side of the membrane, as previously suggested [Tron and Lemesle‐Meunier, 1990; Bruel et al., 1995].

### Clinical Relevance and Future Prospects

The continual advancement of sequencing technologies has allowed the identification of increasing numbers of *mt‐cyb* variations in humans. However, it remains impossible to introduce single mutations into human mtDNA and thus observe any direct genotype–phenotype correlations at the molecular or cellular level. Although the technology exists to produce custom cybrids, such cell lines are likely to contain several mtDNA variations that could contribute to phenotype [Singh et al., 2014]. Also, the process of creating cybrids can introduce irreversible epigenetic changes [Smiraglia et al., 2008], as well as nuclear genome instability and mutation [Singh et al., 2005], further confounding isolating a single mtDNA mutation as contributing factor. Also, under normal circumstances, subtle changes in complex III properties might be missed, and it is only when cells are forced to use alternative metabolic pathways through changes in their cellular environment, or in the presence of inhibitors, as we have shown here, that they are revealed.

Our focus was to use the yeast model system, whose mtDNA is amenable to genetic manipulation, to investigate the direct functional consequences of human MT‐CYB mutations on corresponding complex III properties. Our results reveal that most of the mutations that cause amino‐acid replacements in key catalytic regions of MT‐CYB affected the sensitivity of complex III to drugs and/or complex III activity. Although the MT‐CYB mutations investigated here could exert a more severe effect in yeast cells than in human cells where they might be present at lower heteroplasmy, the potential clinical relevance of the mutations to humans and recommendations for future work are discussed below.

In this study, we showed seven amino‐acid replacements increased the sensitivity of yeast complex III to either atovaquone or clomipramine (mutations corresponding to the human polymorphisms p.Asp171Asn, p.Asp171Gly, p.Asp171Ser, p.Phe18Leu, p.Asn32Ser, p.Gly38Ser, and p.Phe220Leu). This is particularly significant finding for p.Asp171Asn and p.Phe18Leu, as they frequently occur in the normal/healthy individuals, as well as those with disease. p.Asp171Asn and p.Phe18Leu carriers (along with carriers of the other rarer polymorphisms) might respond more strongly to atovaquone and clomipramine, respectively: their complex III would be inhibited at lower doses than the enzyme of controls, which could lead to respiratory chain defect and impaired metabolism, especially in cells that heavily rely on the respiratory chain for their energy supply. Thus, the polymorphisms would be disadvantageous for individuals/patients who are prescribed the drugs for the treatment of malaria/depression, due to the potential side effects. Such side effects are likely to be even more marked in individuals that already harbor mitochondrial/metabolic disorders. The converse would be expected for individuals carrying the rarer polymorphisms that decrease atovaquone/clomipramine sensitivity. As new Q_o_ and Q_i_ site inhibitors are being developed as antimalarial agents [Doggett et al., 2012; Nilsen et al., 2013; Stickles et al., 2015], it would seem pertinent to determine their selectivity and efficacy on p.Asp171Asn‐ or p.Phe18Leu‐carrying human cells.

While enhanced sensitivity of complex III to atovaquone or clomipramine may be a disadvantage for individuals carrying the polymorphism p.Asp171Asn or p.Phe18Leu, it might be an advantageous phenomenon for the selective killing of cancer cells, for example, glioma, in which p.Asp171Asn and p.Phe18Leu are reported. Further investigation into the role of mitochondrial genetic background in governing the potential selectivity/efficacy of chemotherapeutic strategies that target complex III on human cancer cells is therefore warranted. For example, the presence of p.Asp171Asn or p.Phe18Leu could be used as an inclusion criterion for preclinical trials testing either atovaquone or clomipramine on human cancer cells or in rodent models. Retrospective analyses could then be conducted to determine whether p.Asp171Asn/p.Phe18Leu carriers responded better to treatment than those that did not carry the mutation.

Also in this study, we identified three amino‐acid replacements that severely decreased complex III activity, namely, p.Gly136Ser, p.Gly136Asp, and p.Gly142Glu. These mutations, due to their impact on yeast or bacterial complex III, are expected to be severely pathological in humans. This could explain why they are rarely detected in the human population. The few normal, healthy individuals that harbor these mutations could be examples of so‐called “resilient” individuals that despite possessing mutations for severe Mendelian conditions, have no reported clinical manifestations of the indicated disease [Chen et al., 2016]. Samples from these patients could be of interest to scientists who are seeking to discover genetic and environmental modulators that buffer the effects of, in this case, potentially deleterious mitochondrial mutations.

Other mutations (p.Asn32Ser and p.Leu149Met) would result in a milder effect on complex III activity, which could still impact the overall activity of the respiratory chain. These mutations might also be pathogenic, as previously predicted for p.Asn32Ser, a LHON‐associated mutation [Lloyd and McGeehan, 2013].

Finally, several substitutions are not expected to impair complex III activity and are unlikely to be pathogenic: p.Phe18Leu, p.Gly38Ser, p.Gly101Ala, p.Gly101Ser, p.Gly101Asp, p.Thr126Ala, p.Asp171Asn, p.Asp171Gly, p.Asp171Ser, p.Phe220Leu, p.Asp252Glu, and p.Asp252Asn. This is interesting because the frequently occurring polymorphisms p.Asp171Asn and p.Phe18Leu have been proposed to have a pathogenic role in numerous diseases. Through association, p.Asp171Asn has been proposed to cause one of the mildest LHON phenotypes, where patients have difficulty in perceiving hand motion [Johns et al., 1993]. However, results presented here from yeast cells suggest that p.Asp171Asn is unlikely to contribute to LHON in isolation and possibly other co‐occurring mutations, either alone or in combination with p.Asp171Asn, are more likely to be responsible, as suggested previously [Mackey et al., 1996]. Similarly, through association, p.Phe18Leu (and the rarer p.Gly101Asp and p.Asp252Asn) has been proposed to contribute to several diseases, including GBM [Lloyd et al., 2015]. Results presented here in yeast cells suggest p.Phe18Leu and the other mutations are unlikely to be responsible, but could play a role in clomipramine sensitivity.

We show that several human‐associated mutations, when introduced into yeast mtDNA, affect complex III activity and/or drug sensitivity, with no clear relationship between the two parameters. These findings suggest that while complex III mutations could play a greater role in human health and disease than previously thought, they also highlight once again the challenges associated with predicting the functional importance of amino‐acid substitutions in MT‐CYB, presumably due to the complicated cross‐talk that exists between mitochondrial function and the cellular environment. However, the yeast model system provides the opportunity to probe such relations in an uncomplicated genetic background under different physiological conditions, including in the presence of inhibitors. This compendium of new *mt‐cyb* biochemical relationships in yeast presented here will help clinicians and scientists decide which variations to prioritize for future preclinical investigations in humans.

*Disclosure statement*: The authors declare no conflict of interest.

## Supporting information

Disclaimer: Supplementary materials have been peer‐reviewed but not copyedited.

Supp. Figure S1. Location of residues in possible proton routes in the yeast Qo pocket. Yeast cytochrome *b* is colored in blue, stigmatellin in grey, heme *b*l in red, water molecules in black. Residues p.Tyr132, p.Gly137, p.Ser140, p.His253 and p.Glu272 are colored in yellow and the sidechains are shown. Hydrogen bonds are calculated and generated by Chimera, shown by black lines. The figure was drawn using the coordinates 3CX5 of yeast complex III.
Supp. Table S1. Yeast MT‐CYB mutants: complex III activity and sensitivity to inhibitors
Supp. References
