# Supplementary material for: Human Mitochondrial Cytochrome b Variants Studied in Yeast: Not All Are Silent Polymorphisms
Source: Hum Mutat. 2016 Jun 27;37(9):933–41. doi: 10.1002/humu.23024 (PMC5094555; doi:10.1002/humu.23024)
Supplement: Supplementary file 1 — Supp. Figure S1. Location of residues in possible proton routes in the yeast Qo pocket. Yeast cytochrome b is colored in blue, stigmatellin in grey, heme bl in red, water molecules in black. Residues p.Tyr132, p.Gly137, p.Ser140, p.His253 and p.Glu272 are colored in yellow and the sidechains are shown. Hydrogen bonds are calculated and generated by Chimera, shown by black lines. The figure was drawn using the coordinates 3CX5 of yeast complex III. Supp. Table S1. Yeast MT‐CYB mutants: complex III activity and sensitivity to inhibitors Supp. References [file HUMU-37-933-s001.pdf]

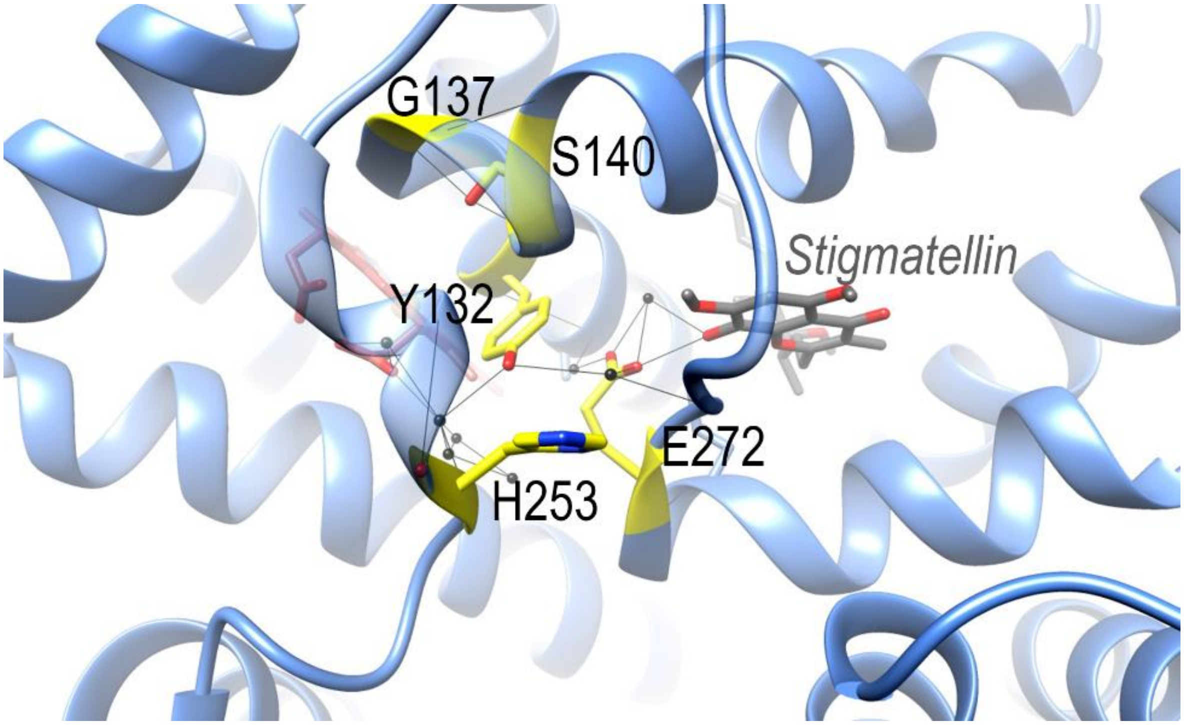

Supp. Figure S1. Location of residues in possible proton routes in the yeast Q_o pocket. Yeast cytochrome *b* is colored in blue, stigmatellin in grey, heme b_l in red, water molecules in black. Residues p.Tyr132, p.Gly137, p.Ser140, p.His253 and p.Glu272 are colored in yellow and the sidechains are shown. Hydrogen bonds are calculated and generated by Chimera, shown by black lines. The figure was drawn using the coordinates 3CX5 of yeast complex III.

Supp. Table S1. Yeast MT-CYB mutants: complex III activity and sensitivity to inhibitors

**Q_o domain : atovaquone sensitivity**			
	**complex III (% control)**	**atovaquone sensitivity (IC_{50} mutant/IC_{50} control)**	**references**
p.Thr127Ile	81	1	(Hill et al. 2003)
CysCysVal $_{133-135}$ ValLeuPro	100	3.5	(Fisher and Meunier 2005)
p.His141Tyr	52	0.5	(Vallières et al. 2013)
p.His141Phe	68	0.4	(Fisher and Meunier 2005)
p.Gly143Ala	87	9	(Fisher and Meunier 2005)
p.Gly143Asp [a]	inactive	nd	(Daldal et al. 1989)
p.Leu150Phe	54	3	(Hill et al. 2003)
p.Ser172Asp	72.3 ± 5.5	1.9	This work
p.Ser172Asn	86.5 ± 9.2	0.9	This work
p.Ser172Gly	110 ± 25.4	1.1	This work
**Q_i domain : clomipramine sensibility**			
	***bc_1* complex activity (s^{-1})**	**clomipramine sensitivity (IC_{50} mutant/IC_{50} control)**	
p.Ile17 (WT)	56 ± 4.6	1	This work
p.Ile17Phe	46 ± 15.1	2.2	This work
p.Asn31Ser	25 ± 0.8	0.15	This work
p.Gly37Ser	47 ± 3	0.4	This work
p.Gly100Asp	42.5 ± 1.6	2.1	This work
IlePheGlyAsp	49 ± 1	2.8	This work
p.Phe225Leu	50.7 ± 2.1	0.6	This work
**Q_o domain: proton pathway**			
	bc_1 complex activity (s^{-1})		
p.Tyr132Phe	23 ± 0.5		This work
p.His253Glu	5 ± 0.3		This work
p.Gly137Arg	17 ± 0.9		This work
p.His253Asp	79.3 ± 5.3		This work
p.His253Glu	85 ± 6		This work
p.His253Asn	78 ± 5		This work

The mutant strains were constructed by biolistic transformation method as described in Materials and Methods, except mutant G137E that was obtained after random mutagenesis (Tron and Lemesle-Meunier 1990). [a] p.Gly143Ala was studied in bacteria bc_1 complex.

The bc_1 complex activity assays (decylubiquinol cytochrome *c* reduction) and determination of inhibitor mid-point titration (IC_{50}) were performed as described in Materials and Methods. For the first six mutants of the table, the data were from the listed publications.

Supp. References

Daldal F, Tokito MK, Davidson E, Faham M. 1989. Mutations conferring resistance to quinol oxidation (Qz) inhibitors of the cytochrome bc_1 complex of *Rhodobacter capsulatus*. EMBO J. 8: 3951–3961.

Fisher N, Meunier B. 2005. Re-examination of inhibitor resistance conferred by Qo-site mutations in cytochrome *b* using yeast as a model system. Pest Manag. Sci. 61: 973–8.

Hill P, Kessl J, Fisher N, Meshnick S, Trumpower BL, Meunier B. 2003. Recapitulation in *Saccharomyces cerevisiae* of cytochrome *b* mutations conferring resistance to atovaquone in *Pneumocystis jiroveci*. Antimicrob. Agents Chemother. 47: 2725–2731.

Tron T, Lemesle-Meunier D. 1990. Two substitutions at the same position in the mitochondrial cytochrome *b* gene of *S. cerevisiae* induce a mitochondrial myxothiazol resistance and impair the respiratory growth of the mutated strains abbeit maintaining a good electron transfer activity. Curr. Genet. 18: 413–419.

Vallières C, Fisher N, Meunier B. 2013. Reconstructing the Q_o site of *Plasmodium falciparum* bc_1 complex in the yeast enzyme. PLoS One 8: e71726.
